# Effect of severity and cause of preoperative anemia on the transfusion rate after total knee arthroplasty

**DOI:** 10.1038/s41598-022-08137-9

**Published:** 2022-03-08

**Authors:** Tae Woo Kim, Hyung Jun Park, Moon Jong Chang, Sang Yoon Kang, Kee Soo Kang, Chong Bum Chang, Seung-Baik Kang

**Affiliations:** 1 Department of Orthopedic Surgery, Seoul National University College of Medicine, Seoul Metropolitan Government-Seoul National University Boramae Medical Center, 20, Boramae-ro 5-gil, Dongjak-gu, Seoul, 07061 South Korea; 2 Department of Orthopaedic Surgery, Seoul National University Bundang Hospital, Seongnam, South Korea; 3 Department of Orthopaedic Surgery, Seoul National University Hospital, Seoul, South Korea; 4 Seoul National University College of Medicine, Seoul, South Korea

**Keywords:** Risk factors, Signs and symptoms

## Abstract

This study aimed to (1) evaluate the preoperative Hb cut-off value for transfusion after unilateral and bilateral staged (1 week apart) TKAs, respectively, and (2) determine whether cause of preoperative anemia can affect transfusion rate after TKA. A total of 951 patients who underwent TKA (unilateral: 605, bilateral staged: 346) from 2016 to 2019 were reviewed retrospectively. Patient demographics, comorbidities, preoperative Hb level, surgery types, and cause of anemia were evaluated as possible risk factors. The cut-off values for preoperative Hb level to reduce transfusion after TKA were evaluated in each surgery type. Preoperative Hb level, surgery type, and cardiac disease were identified as the risk factors for transfusion after TKA, and preoperative Hb levels of 11.8 (AUC 0.88) and 12.8 (AUC 0.76) were the cut-off values for transfusion after unilateral and staged bilateral TKAs, respectively. Although transfusion rate was higher in anemia with iron deficiency (ID) group than anemia without ID group, preoperative Hb level was also lower in anemia with ID group than anemia without ID group. Single use of preoperative Hb level with different cut-offs depending on the surgery types can be useful indicator for preoperative optimization regardless of cause of anemia.

## Introduction

Total knee arthroplasty (TKA) is an efficient surgical option for treating end-stage arthritis of the knee^1–3^. However, perioperative blood loss and consequent hemoglobin (Hb) drop can result in the need for allogeneic transfusion in some patients. Although it can correct anemia effectively, related side effects such as immunologic reaction, disease transmission, and cardiopulmonary complication remain critical concerns^4,5^.

Preoperative Hb level is a well-known and potent risk factor for transfusion after TKA^6–8^. Several guidelines related to patient blood management (PBM) or enhanced recovery after surgery (ERAS) have recommended correction of anemia before TKA using iron or erythropoietin supplementation^9–12^. These efforts to elevate preoperative Hb levels reveal reduced transfusion rates after TKA^13–17^.

However, the appropriate criteria for effective Hb correction before TKA remain controversial. Depending on the anemia definitions, some guidelines use an Hb level of 12.0 g/dL in women and 13 g/dL in men for preoperative correction^18^, while others recommend the Hb level of 13.0 g/dL in both men and women^19^. Although several studies have investigated the Hb cut-off value to predict transfusion after TKA, these studies were limited to unilateral TKA, and tranexamic acid (TXA) was not used in a few others^20,21^.

With preoperative Hb level, cause of anemia also can be considered as a risk factor for transfusion after TKA. Theoretically, hematopoietic response after surgical blood loss can be different depending on the cause of anemia and iron storage status, and it can result in different transfusion rate after TKA. However, whether the cause of anemia can affect transfusion rate after TKA has not been investigated yet.

Therefore, the purpose of this study were to (1) evaluate the preoperative Hb cut-off value for transfusion after unilateral and bilateral staged (1 week apart) TKAs, respectively, in the use of TXA, and (2) determine whether cause of preoperative anemia can affect transfusion rate after TKA.

## Methods

### Study population

**Figure 1:**
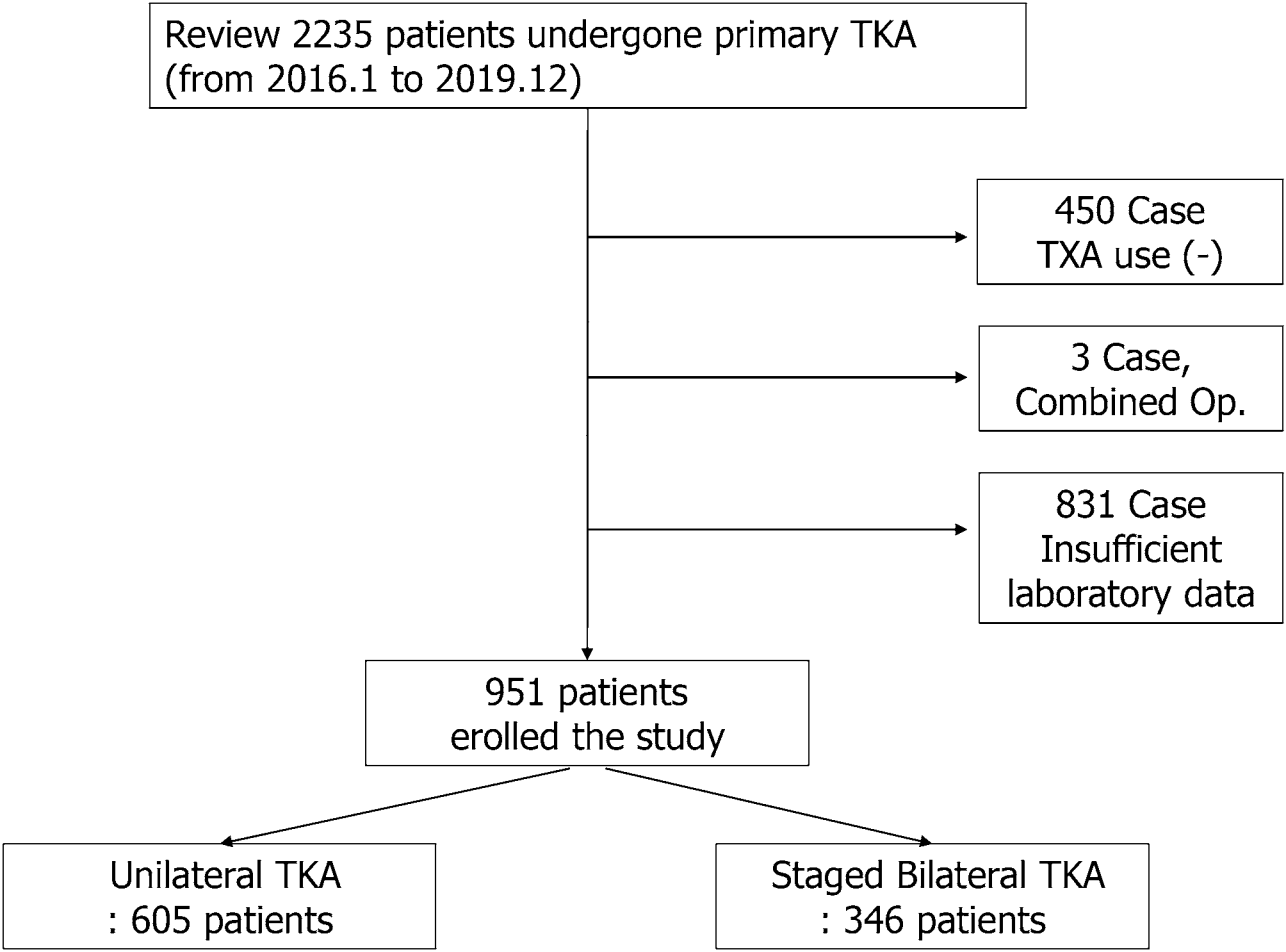
Flow chart of patient enrollment in this study.

We retrospectively reviewed 2238 patients who underwent primary unilateral or bilateral staged TKAs by three experienced arthroplasty surgeons (K.S.B, C.C.B, C.M.J) at a single institution between January 2016 and December 2019. All bilateral staged TKAs were performed at 1-week intervals, and simultaneous bilateral TKAs were not included. Patients who did not use TXA perioperatively, those who underwent combined procedures such as hardware removal, and who did not undergo testing for serum iron profile preoperatively were also excluded. Finally, 951 patients (unilateral TKA, 605; bilateral staged TKA, 346), (K.S.B,350; C.C.B,347; C.M.J,254) were included in this study (Fig. 1). There were 820 women (86.2%) and 131 men (13.8%), and the average age of the study population was 71.7 ± 6.3 years.

### Data collection

Patient demographics including sex, age, body mass index (BMI), and combined comorbidities such as cardiac disease, chronic renal failure, and ASA level were analyzed based on the medical records. Arrhythmia, coronary heart disease, and valve disease were included in cardiac disease, and an estimated glomerular filtration rate less than 60 mL/min was regarded as chronic renal failure. Hb was measured 1 day prior to surgery, immediately after surgery, and on postoperative day (POD) 1, 2, 4, and 6. The maximal Hb drop was calculated by subtracting the lowest postoperative Hb level from the preoperative Hb level. For assessing cause of anemia, preoperative serum iron profiles including ferritin, iron, total iron binding capacity, and transferrin saturation (TS) levels were investigated. Anemia was defined as Hb level below 12.0 g/dL in women and 13 g/dL in men according to the World Health Organization (WHO) guidelines. The cause of anemia was classified as follows^24^: (1) definite iron deficiency anemia (IDA): ferritin < 30 µg/L, (2) IDA combined with anemia of chronic disease (ACD): 30 µg/L < ferritin < 100 µg/L, and TS < 20%, (3) ACD: ferritin > 100 µg/L, and TS < 20%; (4) anemia of other cause (AOC). IDA and IDA + ACD were considered anemia with iron deficiency (ID), and ACD and AOC were considered as anemia without ID. The type of surgery (unilateral or staged bilateral), use of TXA, and postoperative transfusion were also evaluated. This study was approved by the institutional review committee of our hospital, and the requirement for informed consent was waived because of its retrospective nature (Seoul National University Boramae Medical Center Institutional Review Board, No. 20-2020-171).

### Surgical procedures and perioperative management

All surgeries were performed using the cemented posterior-stabilized TKAs with tourniquet application. Using the medial parapatellar approach, femoral cutting was performed using an intramedullary guide, and the intramedullary canal was sealed with a bone plug. Tibial cutting was performed with an extramedullary guide. After closure of the joint capsule, the drain was placed in the subcutaneous layer and removed 1 day after the operation. TXA (1 or 2 g) was administered topically into the joint cavity or intravenously during the surgery. Walker ambulation and continuous passive motion exercises were permitted 1 day after the operation in all patients. Postoperative transfusion was performed when the Hb level was < 7.0 g/dL or < 8.0 g/dL in patients with anemia symptoms. At discharge, oral iron (ferrous sulfate 500 mg/day) was prescribed for 4 weeks in all patients with anemia. Except for tranexamic acid protocol (2 surgeons: 2 g intraarticular, 1 surgeon: 1 g intraarticular + 1 g intravenous), blood management protocol was identical among three surgeons. For the prevention of venous thromboembolism, intermittent pneumatic compression device was used, and aspirin (100 mg/day) was administered for 6 weeks after surgery.

### Data analysis

In univariate analysis, patient demographics, comorbidities including cardiac disease, chronic renal failure, and ASA level, preoperative Hb level, surgery types, and cause of anemia classified based on the iron deficiency were compared between transfusion, and non-transfusion groups to select meaningful variables. Then, multivariate logistic regression was performed to identify risk factors for transfusion after TKA, and determine whether cause of anemia can affect transfusion after TKA. To evaluate the effect of preoperative anemia severity depending on the surgery types, cut-off values of Hb level for transfusion after unilateral, and bilateral TKAs were calculated, respectively.

To analyzed the effect of severity and cause of anemia simultaneously, scatter plot showing the distribution of preoperative Hb level, and iron storage status (ferritin level) between transfusion, and non-transfusion groups were created. Then, transfusion rate, and preoperative Hb levels were compared between anemia with ID, and anemia without ID groups were compared. All method was carried in accordance with relevant guidelines and regulation.

### Statistics

All statistical analyses were performed using SPSS version 26.0 (IBM, Armonk, NY, USA). The data are presented as means and standard deviations for continuous variables. For univariate analysis between transfusion and non-transfusion groups, the Chi-square test and Fisher’s exact test were used for categorical variables and t-test was used for continuous variables. Statistical significance was set at *p* < 0.05. For variables that showed significant differences in the univariate analysis, multivariate logistic regression was performed to identify risk factors for transfusion. For determining the cut-off value of each parameter, the sensitivity, specificity, and estimated area under the curve (AUC) of the receiver operating characteristics (ROC) curve were analyzed.

## Results

**Figure 2:**
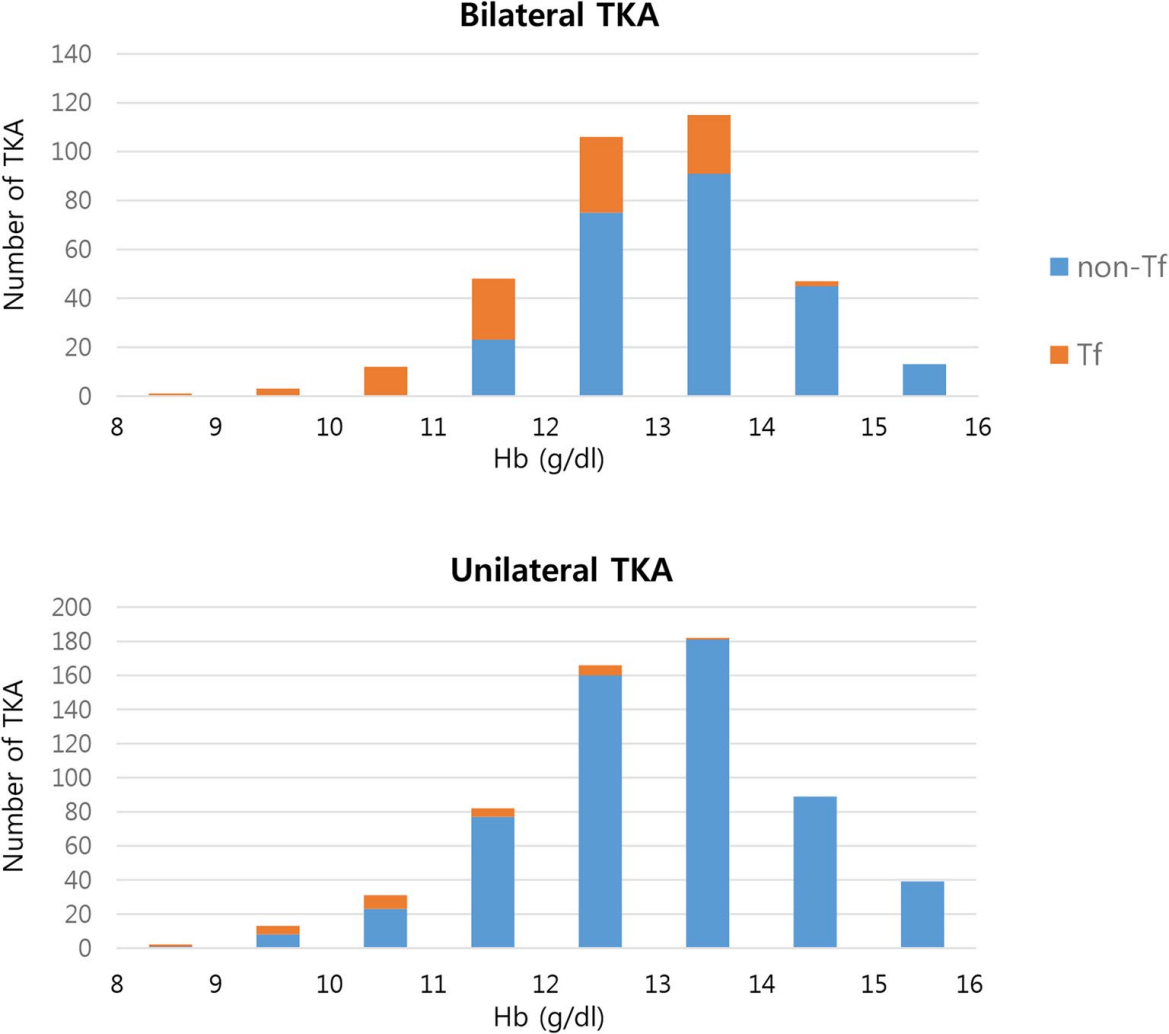
Comparison of transfusion rate between unilateral and bilateral staged (1 week apart) total knee arthroplasty (TKA). In bilateral staged TKA, transfusion rate in non-anemic patients was higher than that of unilateral TKA.

A total of 125 patients (125/951, 13.1%) underwent transfusion after primary TKA. The transfusion rate was 4.3% (26/605 patients) in unilateral TKA and 28.6% (99/346 patients) in bilateral staged (1 week apart) TKA (Fig. 2). However, there was no difference in transfusion rate among three surgeons (K.S.B: 12.1%, C.M.J:12.3%, C.C.B: 11.9%, p > 0.05). The mean maximal Hb drop was 3.6 ± 1.3 g/dL, and maximal Hb drop was significantly increased in transfusion group compared to non-transfusion group (transfusion: 4.3 ± 1.2 g/dL, non-transfusion: 3.26 ± 1.3 g/dL, p < 0.05).

In the univariate analysis, the transfusion group showed significantly older age, higher female proportion, increased comorbidities including cardiac disease, chronic renal failure, decreased preoperative Hb level, increased iron deficiency, and more bilateral staged TKA cases (all p = 0.001). However, BMI and ASA levels were not related to transfusion after TKA (Table 1).

**Table 1:** Patient demographics and preoperative factors.

Preoperative factors	Total (n = 951)	Transfusion (n = 125)	Non-transfusion (n = 826)	*p* value
Age at surgery (SD)	71.6 (6.3)	73.3 (6.7)	71.4 (6.2)	0.001*
Gender (female) (n, %)	820 (85)	120 (96)	700 (85)	0.001*
BMI (kg/m^2^) (SD)	27.5 (14.7)	26.5 (3.5)	27.6 (15.7)	0.440
**Comorbidities (n, %)**				
Cardiac disease	91 (9.6)	22 (17.6)	69 (8.4)	0.001*
Chronic renal failure	90 (9.4)	21 (17.3)	69 (8.4)	0.003*
**ASA**				0.072
Class 1	111	14 (11.2)	97 (11.7)	
Class 2	756	93 (74.4)	663 (80.3)	
Class 3	84	18 (14.4)	66 (8)	
Preoperative Hb (g/dL) (SD)	12.9 (1.3)	11.9 (1.4)	13.1 (1.2)	0.001*
**Cause of anemia (n, %)**				
Total	214	62	152	0.001*
IDA	71	27 (43.5)	44 (28.9)	
IDA + ACD	47	15 (24.2)	32 (21.1)	
ACD	14	2 (3.2)	12 (7.9)	
AOC	82	18 (29)	64 (42.1)	
Anemia with ID	118	42 (67.7)	76 (43)	0.004*
**Surgery types (n, %)**				0.001*
Unilateral	605	26 (20.8)	579 (70.1)	
Staged bilateral	346	99 (79.2)	247 (29.9)	

The data are presented as means and standard deviations for continuous variables. Statistical significance was set at *p* < 0.05*. *BMI* body mass index, *ASA* American society of anesthesiologistas, *Hb* hemoglobin, *ID* iron deficiency, *IDA* iron deficiency anemia, *ACD* anemia of chronic disease, *AOD* anemia of other cause.

In the multivariate logistic analysis, preoperative Hb level (odds ratio 2.25, 95% confidence interval [CI] 1.64–3.10), cardiac disease (odds ratio 0.28, 95% CI 0.14–0.55), and type of surgery (odds ratio 0.06, 95% CI 0.03–0.10) were identified as independent predictors for transfusion after TKA (Table 2). Different from the univariate analysis results, cause of anemia based on the iron deficiency did not affect transfusion after TKA in multivariate logistic regression.

**Table 2:** Multivariate logistic regression of transfusion after TKA.

Preoperative factors	Odds Ratio	Lower 95% of CI	Upper 95% of CI	*p* value
Age at surgery	0.99	0.95	1.02	0.581
Gender (female)	1.7	0.58	4.92	0.329
**Comorbidities**				
Cardiac disease	0.28	0.14	0.55	0.000*
Chronic renal failure	1.0	0.49	2.03	0.999
Preoperative Hb (g/dL)	2.26	1.64	3.10	0.000*
**Cause of anemia**				
IDA	0.88	0.34	2.26	0.790
IDA + ACD	0.52	0.09	3.03	0.471
ACD	0.41	0.14	1.16	0.093
AOC	0.66	0.21	2.01	0.485
Anemia with ID	0.90	0.51	1.60	0.728
**Surgery types**				
Unilateral/staged bilateral	0.06	0.03	0.10	0.000*

The data are presented as means and standard deviations for continuous variables. Statistical significance was set at *p* < 0.05*. *BMI* body mass index, *ASA* American society of anesthesiologistas, *Hb* hemoglobin, *ID* iron deficiency, *IDA* iron deficiency anemia, *ACD* anemia of chronic disease, *AOD* anemia of other cause.

**Figure 3:**
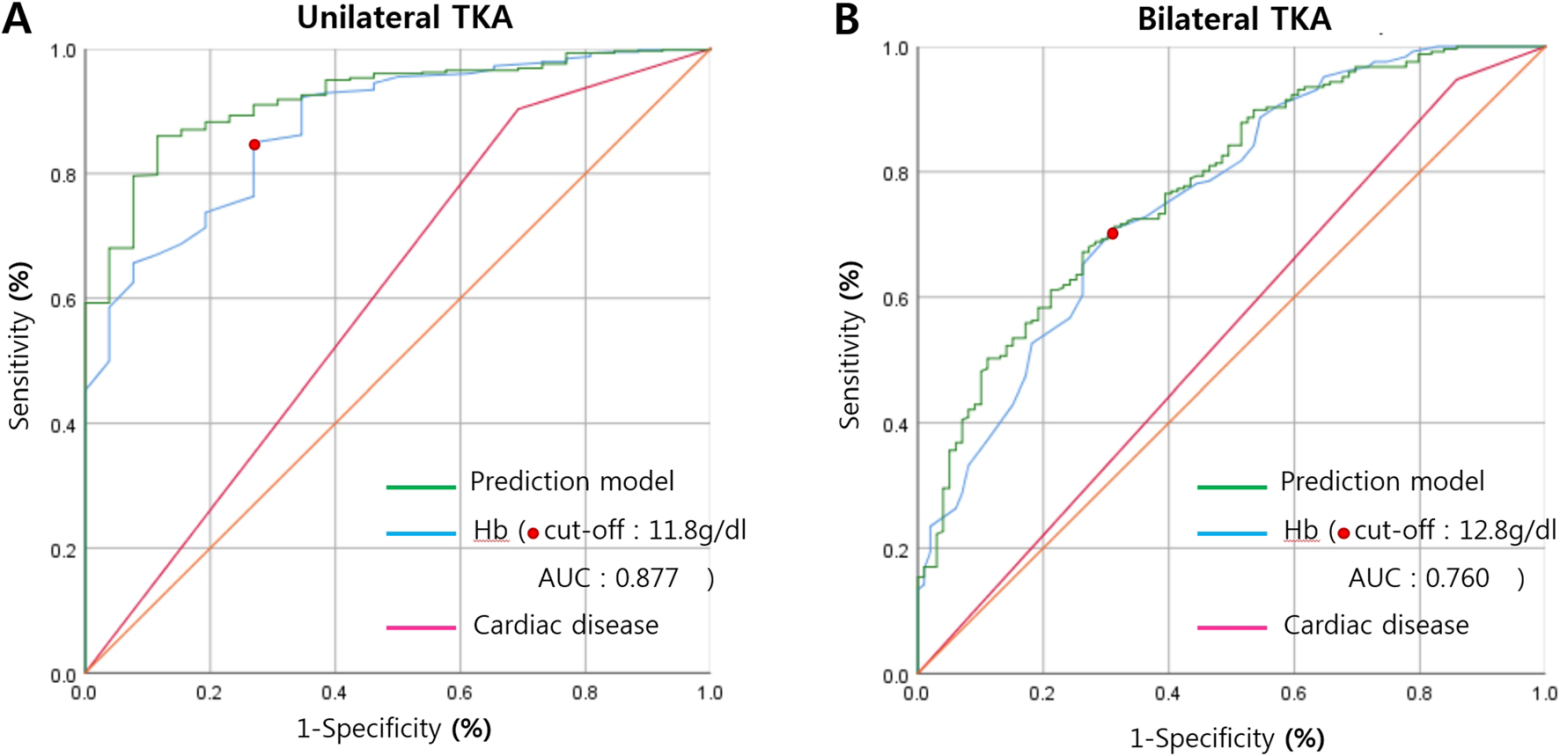
(**A**) The receiver operating characteristic curve of preoperative hemoglobin level, and cardiac disease in unilateral total knee arthroplasty (TKA). This curve indicate that the cut off value of preoperative hemoglobin as 11.8 g/dL (red dot, AUC 0.877, sensitivity 85%, specificity 73.1%). (**B**) The receiver operating characteristic curve of preoperative hemoglobin level, and cardiac disease in bilateral staged (1 week apart) TKA. This curve indicate that the cut off value of preoperative hemoglobin as 12.8 g/dL (red dot, AUC 0.760, sensitivity 71%, specificity 70.2%).

The ROC curves illustrated that preoperative Hb level can be a useful parameter to predict transfusion after TKA. In unilateral TKA, the AUC for preoperative Hb level was 0.877, and the cut-off value was determined to be 11.8 g/dL, with a sensitivity of 85% and specificity of 73.1% (Fig. 3A). In staged bilateral TKA, the AUC for preoperative Hb level was 0.760, and the cut-off value was determined to be 12.8 g/dL, with a sensitivity of 71% and specificity of 70.2% (Fig. 3B).

**Figure 4:**
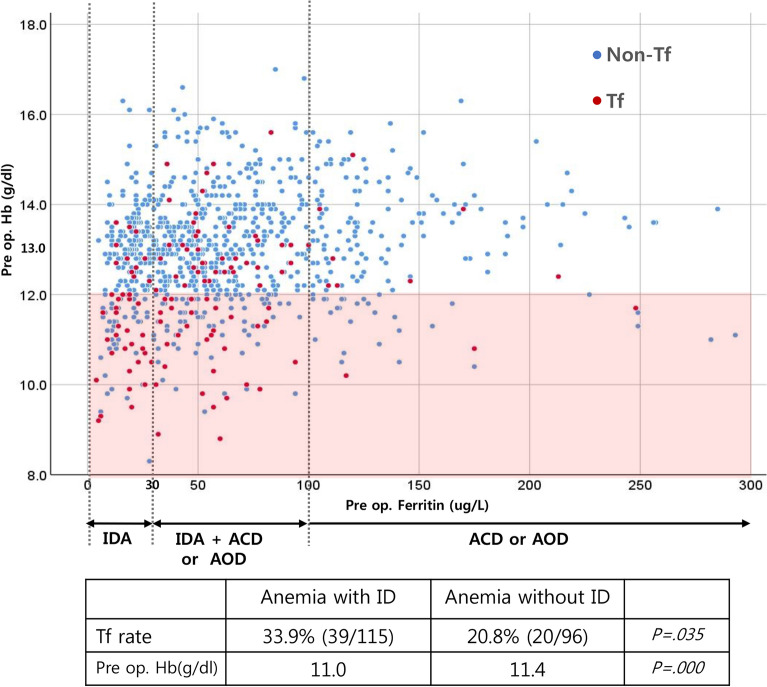
Scatter plot showing the distribution of preoperative Hb level, and iron storage status (ferritin level) between transfusion, and non-transfusion groups. In anemia with iron deficiency (ID), significantly decreased preoperative Hb level, and higher transfusion rate than anemia without ID were observed, this finding is well illustrated in the scatter plot.

Preoperative anemia was observed in 213 patients (22.5%), and among the patients with anemia, IDA, IDA + ACD, ACD, and AOC accounted for 33%, 22%, 6.5%, and 38.5%, respectively. Anemia with and without iron deficiency (ID) was seen in 55% and 45% of cases, respectively. Although transfusion rate was significantly higher in anemia with ID group than anemia without ID group (33.9%, 20.8%, p = 0.035), preoperative Hb level was also significantly lower in anemia with ID group than anemia without ID group (11.0, 11.4, p = 0.000) (Fig. 4).

## Discussion

The important finding of this study was that preoperative Hb level, type of surgery, and cardiac disease were defined as risk factors for transfusion after TKA. In the use of TXA, preoperative Hb levels of 11.8 g/dL and 12.8 g/dL were the cut-off values for transfusion after unilateral and bilateral staged (1 week apart) TKAs, respectively. Despite higher transfusion rate in anemia with ID compared to anemia without ID, when considered with preoperative Hb level, cause of anemia did not affect transfusion after TKA.

In this study, preoperative Hb level, type of surgery, and cardiac disease were defined as risk factors for transfusion after TKA, and these results were well matched with those of previous studies. Jo et al.^7^ reported Hb level, type of surgery, use of TXA, platelet count, age, and body weight as risk factors for transfusion after TKA. Hu et al.^8^ also presented the Hb level, age, BMI, and coronary heart disease as risk factors. However, unlike previous studies that tried to develop a prediction model including different variables, this study minimized variables to Hb level and cardiac disease by excluding TXA non-users and evaluating predictors separately depending on the surgery type. Through these efforts, a single use of preoperative Hb level revealed considerable predictive power for transfusion after TKA.

In the present study, the cut-off value of preoperative Hb level for transfusion after unilateral TKA was 11.8 g/dL, and this result was similar to or lower than that of previous studies. Yang et al.^20^ recommended preoperative Hb cut-off values of 12.4 g/dL for those aged above 70 years and 12.1 g/dL for those aged below 70 to predict transfusion after unilateral TKA with the use of TXA, and this was similar to our results. In contrast, Maempel et al.’s study^21^ that did not use TXA suggested threshold levels of preoperative Hb as 13.75 g/dL for men and 12.75 g/dL for women. The use of TXA during TKA may have lowered the preoperative Hb cut-off value to predict transfusion after TKA. Our results suggest that the current criteria for preoperative Hb correction (12–13 g/dL) can be slightly lowered in unilateral TKA with the use of TXA.

In contrast, the cut-off value of preoperative Hb level for transfusion after staged bilateral TKA was 12.8 g/dL in this study. To the best of our knowledge, this is the first documentation of the cut-off value for preoperative Hb optimization in bilateral TKA. This criterion for preoperative Hb correction is higher than the popularly used WHO anemia criteria, especially in female patients. However, considering that the transfusion rate (28%) was still high in staged bilateral TKA regardless of TXA use, a higher triggering level of preoperative Hb may be necessary in bilateral TKA compared to unilateral TKA.

Another remarkable finding of this study was that the cause of anemia did not affect the transfusion rate after TKA. Anemia with ID showed significantly decreased preoperative Hb level compared to anemia without ID, and that may have led to a higher transfusion rate in anemia with ID than anemia without ID. Therefore, single use of preoperative Hb level can be sufficient indicator for prediction of transfusion after TKA regardless of cause of preoperative anemia. Although IDA is a well-known risk factor for transfusion after TKA, and iron supplementation has been popularly used to optimize preoperative Hb levels before TKA^22^, it was found that anemia without iron deficiency, such as ACD or AOC, also accounts for 45% of preoperative anemia in patients undergoing TKA; thus, an appropriate correction method for ACD or AOC should also be considered for optimal preoperative optimization.

There are some limitations to this study. First, simultaneous bilateral TKA was not included in this study, and the cut-off value of preoperative Hb level can be different between simultaneous and staged TKA. Nevertheless, the importance of different triggering criteria depending on TKA surgery types for effective preoperative Hb optimization can be a meaningful result of this study. Second, the administration method of TXA was different among the three operators. Two operators locally injected 2 g TXA into the joint cavity; the other operator locally injected 1 g TXA into the joint cavity and then added the 1 g TXA intravenously during the operation. However, the total amount of TXA was not different between the three operators, and several meta-analyses reported comparable transfusion rates between intra-articular and intravenous TXA injections^23^. Therefore, the effect of different TXA administration methods between operators might be minimal in this study.

## Conclusion

Despite higher transfusion rate in anemia with ID compared to anemia without ID, when considered with preoperative Hb level, cause of anemia did not affect transfusion after TKA. Single use of preoperative Hb level with different cut-offs depending on the surgery types can be useful indicator for preoperative optimization regardless of cause of anemia.

## References

[1] Callaghan JJ (2015). What can be learned from minimum 20-year followup studies of knee arthroplasty?. Clin. Orthop. Relat. Res..

[2] Ritter MA (2016). Twenty-five-years and greater, results after nonmodular cemented total knee arthroplasty. J. Arthroplasty.

[3] Polascik BW, Bin Abd Razak HR, Chong HC, Lo NN, Yeo SJ (2018). Acceptable functional outcomes and patient satisfaction following total knee arthroplasty in asians with severe knee stiffness: A matched analysis. Clin. Orthop. Surg..

[4] Stainsby D (2006). Serious hazards of transfusion: A decade of hemovigilance in the UK. Transfus. Med. Rev..

[5] Shander A, Javidroozi M, Ozawa S, Hare GM (2011). What is really dangerous: Anaemia or transfusion?. Br. J. Anaesth..

[6] Owens J, Otero JE, Noiseux NO, Springer BD, Martin JR (2020). Risk factors for post-operative blood transfusion following total knee arthroplasty. Iowa Orthop. J..

[7] Jo C (2020). Transfusion after total knee arthroplasty can be predicted using the machine learning algorithm. Knee Surg. Sports Traumatol. Arthrosc..

[8] Hu C (2020). Development and validation of a nomogram to predict perioperative blood transfusion in patients undergoing total knee arthroplasty. BMC Musculoskelet. Disord..

[9] Wainwright TW (2020). Consensus statement for perioperative care in total hip replacement and total knee replacement surgery: Enhanced Recovery After Surgery (ERAS((R))) Society recommendations. Acta Orthop..

[10] Hu ZC (2019). An enhanced recovery after surgery program in orthopedic surgery: A systematic review and meta-analysis. J. Orthop. Surg. Res..

[11] Muñoz M (2017). International consensus statement on the peri-operative management of anaemia and iron deficiency. Anaesthesia.

[12] Soffin EM, YaDeau JT (2016). Enhanced recovery after surgery for primary hip and knee arthroplasty: A review of the evidence. Br. J. Anaesth..

[13] Biboulet P (2020). Preoperative erythropoietin within a patient blood management program decreases both blood transfusion and postoperative anemia: A prospective observational study. Transfusion.

[14] Chan PK (2020). Blood transfusions in total knee arthroplasty: A retrospective analysis of a multimodal patient blood management programme. Hong Kong Med. J..

[15] Morgan PN (2018). Implementation of a patient blood management program in an Australian private hospital orthopedic unit. J. Blood Med..

[16] Heschl M (2018). The efficacy of pre-operative preparation with intravenous iron and/or erythropoietin in anaemic patients undergoing orthopaedic surgery: An observational study. Eur. J. Anaesthesiol..

[17] Bedair H, Yang J, Dwyer MK, McCarthy JC (2015). Preoperative erythropoietin alpha reduces postoperative transfusions in THA and TKA but may not be cost-effective. Clin. Orthop. Relat. Res..

[18] Kotzé A, Carter LA, Scally AJ (2012). Effect of a patient blood management programme on preoperative anaemia, transfusion rate, and outcome after primary hip or knee arthroplasty: A quality improvement cycle. Br. J. Anaesth..

[19] Muñoz M (2018). An international consensus statement on the management of postoperative anaemia after major surgical procedures. Anaesthesia.

[20] Yeh JZ (2016). Preoperative haemoglobin cut-off values for the prediction of post-operative transfusion in total knee arthroplasty. Knee Surg. Sports Traumatol. Arthrosc..

[21] Maempel JF, Wickramasinghe NR, Clement ND, Brenkel IJ, Walmsley PJ (2016). The pre-operative levels of haemoglobin in the blood can be used to predict the risk of allogenic blood transfusion after total knee arthroplasty. Bone Jt. J..

[22] Theusinger OM, Leyvraz PF, Schanz U, Seifert B, Spahn DR (2007). Treatment of iron deficiency anemia in orthopedic surgery with intravenous iron: Efficacy and limits: A prospective study. Anesthesiology.

[23] Mi B (2017). Intra-articular versus intravenous tranexamic acid application in total knee arthroplasty: A meta-analysis of randomized controlled trials. Arch Orthop. Trauma Surg..

[24] Jans O (2018). Iron deficiency and preoperative anaemia in patients scheduled for elective hip and knee arthroplasty—An observational study. Vox Sang.

